# Commentary on: Evaluating the Quality of Systematic Reviews and Meta-analyses About Breast Augmentation Using AMSTAR

**DOI:** 10.1093/asjof/ojab023

**Published:** 2021-06-11

**Authors:** Daniel J Gould

**Affiliations:** University of Southern California, Los Angeles, CA, USA

A Measurement Tool to Assess Systematic Reviews (AMSTAR) system for reviewing the actual clinical recommendations from systematic reviews is a good approach to identify high-quality recommendations for clinical treatment^1^ and has been externally validated.^2^ This system was developed to help summarize the many different instruments that assess the value of the findings of systematic reviews and represents a powerful developmental leap compared with the original instruments developed by Oxman and Guyatt and Sacks et al.^3-5^ Meta-analysis (proposed by Sacks et al.) has long been the most powerful instrument when coupled with systematic reviews for reporting findings; however, there have been many new tools designed to extract meaning from systematic reviews, many of them are lengthy with over 20 criteria for utilization.^5^The bulkiness of these instruments makes them difficult to use, while AMSTAR has 11 criteria, and they are validated, allowing a streamlined and reproducible approach.

This type of analysis of the plastic surgery literature has been conducted before within hand surgery and has proven valuable.^2,6^ Those authors demonstrated the fact that more systematic reviews were being written and that the quality of the findings was increasing, and if a similar study was conducted for all of the aesthetic literature, there may be some valuable findings with similar trends. It is not a surprise that systematic reviews that followed the Preferred Reporting Items for Systematic Reviews and Meta-Analyses (PRISMA) guidelines have improved AMSTAR scores. This makes sense because the 2 have overlapping criteria. Reporting that criteria is in itself interesting because it does show that the AMSTAR instrument is valid. Unfortunately, there were not many high-value reviews in the literature, and I agree with the authors that there is a need for more high-quality data and for systematic reviews within this subject.

In all cosmetic research, there should be a concerted effort to improve the clinical guidance for outcomes and to cultivate better data to improve patient outcomes. In order for practitioners to keep up with rapidly changing practices in plastic surgery, we need excellent systematic reviews on subjects to clarify the clinical key points and evidence for practice. The truth is that systematic reviews require good primary data in order to have impact. Non-blinded studies can be used but offer less power in their conclusions. I myself have seen that trend, in several systematic reviews, including one published on subfascial breast augmentation not included in this study.^7^ In our study, we found data on thousands of patients, but the quality of the published results was incomplete. That does not mean that we could not learn from the data and draw conclusions for patient care and future studies. We need to continue to innovate and improve the quality of our retrospective and prospective studies while utilizing the data that already exist in the literature.

For all systematic reviews, journals now not only require adherence to PRISMA or other similar guidelines but also require AMSTAR reporting of the actual conclusions in the *Aesthetic Surgery Journal*. We can continue to use Forest plots and those types of analyses to examine the power of the studies and their conclusions, but we do need improved instruments to examine the actual conclusions of these articles. These key criteria will help readers assess the value of the recommendations of the studies, as reported here; in this article, there is an excellent table of the clinically relevant findings of the systematic reviews which should affect practice, education, testing, and outcomes monitoring.^8^This article does a nice job of summarizing the conclusions of the systematic reviews on breast augmentation and providing real, powered clinically relevant data for surgeons and patients. Yuan et al should be applauded for bringing this well-validated instrument into the aesthetic research space to help modernize the research in this area and to prepare our specialty for the future of validated scientific research.^8^

With time, AMSTAR will continue to grow as the most commonly utilized metric for clinically validated findings of reviews and may become required for all clinical journals for systematic reviews.
